# (η^4^-Tri­methyl­ene­methane)(1,1,1-tris­{[bis­(4-meth­oxy­phen­yl)phosphan­yl]meth­yl}ethane)ruthenium(II) diethyl ether hemisolvate

**DOI:** 10.1107/S2414314625005243

**Published:** 2025-06-17

**Authors:** Carolin A. M. Stein, Elisabetta Alberico, Anke Spannenberg, Matthias Beller, Henrik Junge

**Affiliations:** a Leibniz-Institut für Katalyse e V Albert-Einstein-Str 29a 18059 Rostock Germany; b Istituto di Chimica Biomolecolare, CNR, tr. La Crucca 3, 07100 Sassari, Italy; University of Aberdeen, United Kingdom

**Keywords:** crystal structure, ruthenium, tri­methyl­ene­methane ligand, *p*-anisyl-triphos ligand

## Abstract

The title compound, [Ru(C_47_H_51_O_6_P_3_)(C_4_H_6_)]·0.5C_4_H_10_O, consists of an Ru^II^ atom coordinated by 1,1,1-tris­{[bis­(4-meth­oxy­phen­yl)phosphan­yl]meth­yl}ethane in κ^3^-coordination mode and an η^4^-coordinating tri­methyl­ene­methane ligand. The complex mol­ecule is co-crystallized with a diethyl ether solvent mol­ecule. A half diethyl ether mol­ecule was considered, whereas additional disordered solvent mol­ecules were removed from the diffraction data with the SQUEEZE procedure in *PLATON* [Spek (2015 ▸). *Acta Cryst*. C**71**, 9–18].

## Abstract 2

In the title complex, the Ru^II^ atom is coordinated by a 1,1,1-tris­{[bis­(4-meth­oxy­phen­yl)phosphan­yl]meth­yl}ethane ligand in κ^3^-coordination mode and an η^4^-coordinating tri­methyl­ene­methane ligand.

## Graphical abstract

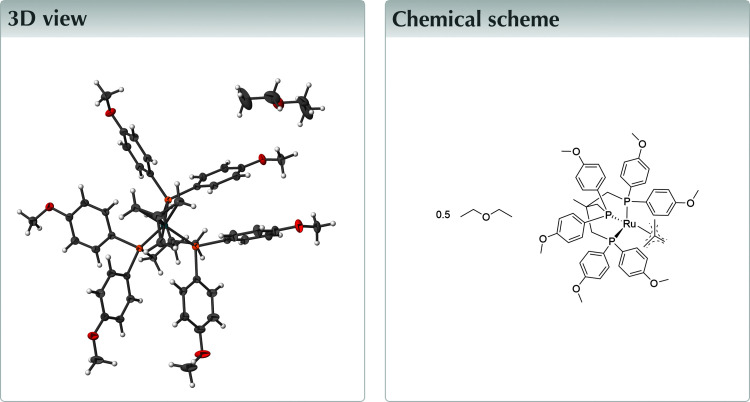

## Structure description

Ruthenium complexes of the general formula [Ru^II^(Ar-triphos)(TMM)] (Ar-triphos = 1,1,1-tris­(di(ar­yl)phosphino-meth­yl)ethane with aryl = *e.g*. phenyl, 4-methyl­phenyl, 3,5-di­methyl­phenyl; TMM = η^4^-tri­methyl­ene­methane) have been successfully applied as catalyst precursors in the hydrogenation of challenging substrates such as carb­oxy­lic esters, amides, carb­oxy­lic acids, carbonates and urea derivatives (vom Stein *et al.*, 2014 ▸) as well as in the hydrogenation of CO_2_ (Wesselbaum *et al.*, 2012 ▸, 2015 ▸). The performance of the title compound, [Ru^II^(*p*-anisyl-triphos)(TMM)], either as pre-formed complex or formed *in situ*, in these transformations is not reported (Maeda *et al.*, 2010 ▸; the synthesis of the ligand *p*-anisyl-triphos is reported in the patent describing the ruthenium homogeneously catalysed hydrogenation of lactones and carb­oxy­lic acid esters in the liquid phase). On the other hand, as part of our inter­est in the design of efficient homogeneous catalytic processes based on non-noble metals, some of us have explored the application of cobalt complexes of Ar-triphos ligands, including *p*-anisyl-triphos, in the hydrogenation of CO_2_ (Scharnagl *et al.*, 2019 ▸) and carbonates (Ferretti *et al.*, 2019 ▸) to methanol as well as in the reductive alkyl­ation of anilines with carb­oxy­lic acids (Liu *et al.*, 2018 ▸) and the de­oxy­genative hydrogenation of amides to amines (Papa *et al.*, 2020 ▸). Xu, Wang, Shi and coworkers have used Co/Ar-triphos complexes as catalysts for the transformation of levulinic acid and amines into pyrrolidines and pyrrolidinones using hydrogen (Pan *et al.*, 2022 ▸). In the latter three synthetic applications (Liu *et al.*, 2018 ▸; Papa *et al.*, 2020 ▸; Pan *et al.*, 2022 ▸), among those tested, the use of the *p*-anisyl-triphos ligand afforded the best results.

**Figure 1:**
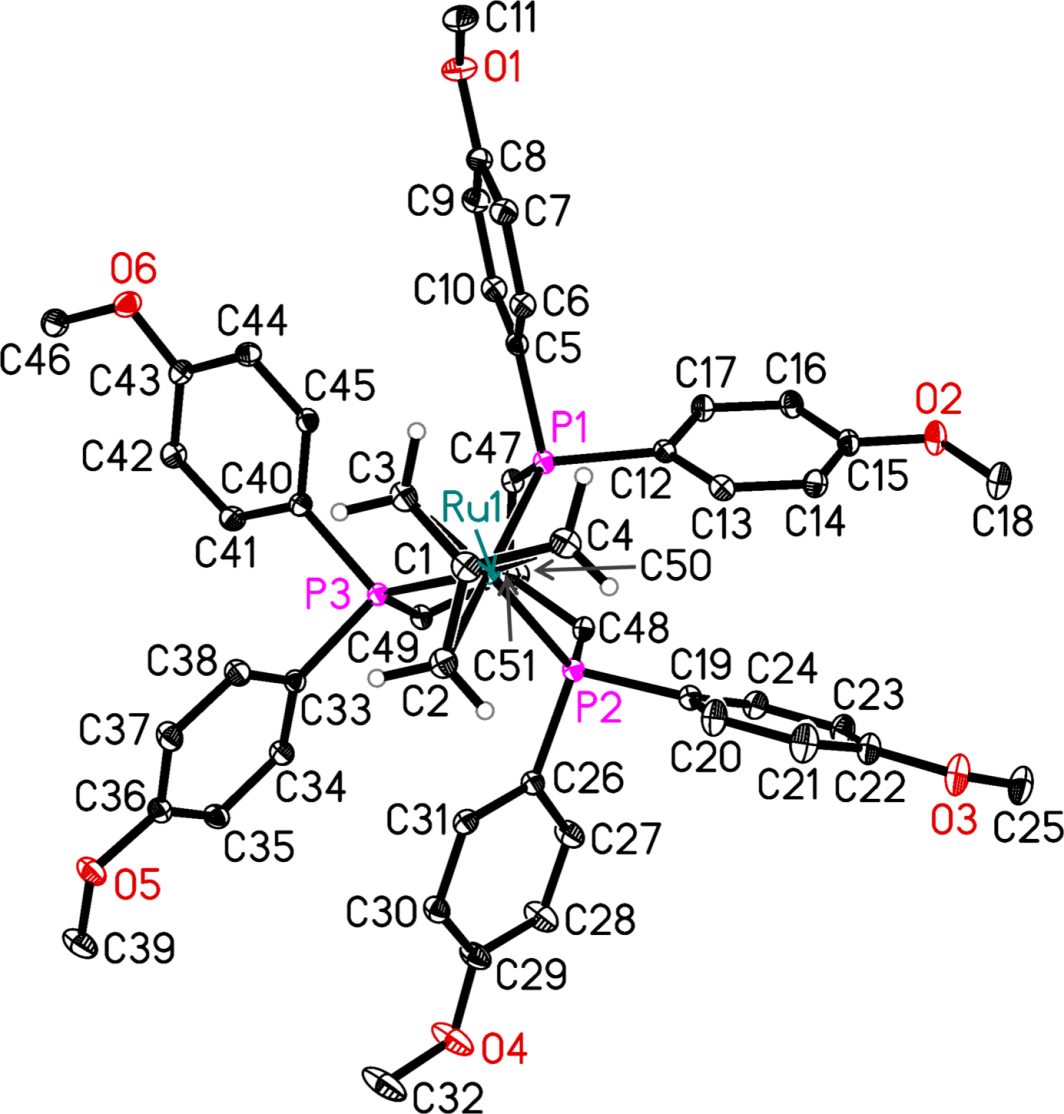
The mol­ecular structure of the title compound without the solvent. Displacement ellipsoids correspond to 50% probability. Hydrogen atoms except those attached to C2, C3 and C4 are omitted for clarity.

We therefore deemed it of inter­est to synthesize the title ruthenium complex to test it as a catalyst precursor. Structural analysis of the isolated complex provided further insights into its coordination chemistry. In the crystal, the three phospho­rus atoms of the neutral C_47_H_51_O_6_P_3_ ligand coordinate to ruthenium generating a facially capped κ^3^ complex. The metal coordination sphere is completed by an η^4^-tri­methyl­ene­methane C_4_H_6_^2–^ dianion acting as a 6 e^−^ donor (Fig. 1 ▸). The title complex exhibits clear asymmetry in both the different Ru—P bond lengths and the corresponding P—Ru—P angles. Whilst the Ru—P1 [2.2787 (6) Å] and Ru—P3 [2.2780 (6) Å] bond lengths are nearly identical, the Ru—P2 bond [2.2988 (5) Å] is significantly elongated. Some asymmetry was also observed in related Ru^II^(Ar-triphos)(TMM) complexes: for Ar = Ph [2.2861 (15), 2.2842 (15), 2.2679 (15) Å (vom Stein *et al.*, 2013 ▸); 2.2893 (5), 2.2720 (5), 2.2885 (5) Å (Savourey *et al.*, 2014 ▸)]; Ar = 3,5-di­methyl­phenyl [2.2812 (8), 2.2907 (7), 2.2749 (8) Å (Meuresch *et al.*, 2016 ▸)]. Notably, another complex known in the literature with Ar = 4-methyl­phenyl has a highly symmetric structure: this complex crystallizes in the trigonal space group *R*3 with only one third of the mol­ecule in the asymmetric unit with Ru—P1 = 2.2757 (6) Å (Meuresch *et al.*, 2016 ▸). The uneven coordination of the 4-meth­oxy-triphos ligand in the title complex is also evident in the bond angles about the metal atom: P1—Ru—P2 = 87.511 (19); P2—Ru—P3 = 87.931 (17); P3—Ru—P1 89.759 (17)°.

The TMM ligand adopts a pyramidal shape with a shorter bond length of the central carbon atom to ruthenium [Ru—C1 = 2.0674 (18) Å] than the terminal carbon atoms [Ru—C2 = 2.245 (2), Ru—C3 = 2.2249 (18), Ru—C4 = 2.263 (2) Å]. The three C—C bonds in the TMM unit are indistinguishable: 1.431 (3), 1.433 (3) and 1.435 (3) Å, which confirms the delocalization of the electrons within this ligand. This is also observed in the related [Ru^II^(Ar-triphos)(TMM)] complexes mentioned above. In solution, at room temperature, the complex exhibits a higher degree of symmetry: in the ^31^P{^1^H} NMR spectrum, a singlet at δ = 31.4 ppm is observed, indicating the spectroscopic equivalence of the three phospho­rus atoms, low field shifted compared to the free ligand, which resonates at δ = −30.4 ppm. For the TMM anion, a singlet is present in the ^1^H{^31^P} NMR spectrum at δ = 1.55 ppm for the six equivalent methyl­ene protons, while the corresponding three methyl­ene carbon atoms all resonate at 42.5 ppm in the ^13^C{^1^H,^31^P} NMR spectrum. The connecting quaternary carbon atom resonates at 107.0 ppm.

The title Ru complex is co-crystallized with diethyl ether solvent mol­ecules. A half diethyl ether mol­ecule per complex mol­ecule was considered, whereas the contribution of additional disordered solvent mol­ecules to the intensity data was removed from the diffraction data with the SQUEEZE procedure in *PLATON* (Spek, 2015 ▸).

## Synthesis and crystallization

The synthesis and isolation of the title complex were performed under an inert atmosphere (argon) with exclusion of air. Toluene, *n*-pentane, diethyl ether and di­chloro­methane were supplied by a solvent purification system and stored over 3 Å mol­ecular sieves. Acetone, 99.8%, Extra Dry, AcroSeal™, was purchased from Thermo Scientific Chemicals. [Bis(2-methyl­all­yl)(1,5-cyclo­octa­diene)ruthenium(II)] was purchased from Strem and used as received. 1,1,1-Tris{[bis­(4-meth­oxy­phen­yl)phosphan­yl]meth­yl}ethane, *p*-anisyl-triphos, was prepared following a published procedure (Wesselbaum *et al.*, 2015 ▸).

^1^H NMR spectra were obtained at 300 MHz (Bruker AV-300), ^13^C{^1^H} NMR spectra were obtained at 75 MHz and ^31^P{^1^H} NMR spectra were obtained at 121 MHz. NMR chemical shifts are reported in ppm downfield from TMS and were referenced to the residual proton resonance and the natural abundance ^13^C resonance of the solvents. ^31^P NMR chemical shifts are reported in ppm downfield from H_3_PO_4_ and referenced to an external 85% solution of H_3_PO_4_.

To a clear colorless solution of *p*-anisyl-triphos (316 mg, 0.39 mmol, 1.1 eq.) in toluene (15 ml), [bis­(2-methyl­all­yl)(1,5-cyclo­octa­diene)ruthenium(II)] (226 mg, 0.36 mmol, 1.0 eq.) was added in one portion. The resulting clear green solution was refluxed for 18 h (overnight). Upon heating the solution became dark. At the end of the specified reaction time, the solution was clear and dark red. It was concentrated *in vacuo* to about 5 ml and *n*-pentane was added to induce precipitation. The dark brownish solid was collected and further washed with acetone thus leaving a gray-greenish solid (140 mg, 40% yield). Crystals suitable for X-ray diffraction were obtained by diffusion of diethyl ether into a solution of the title compound in di­chloro­methane.

**Figure 2:**
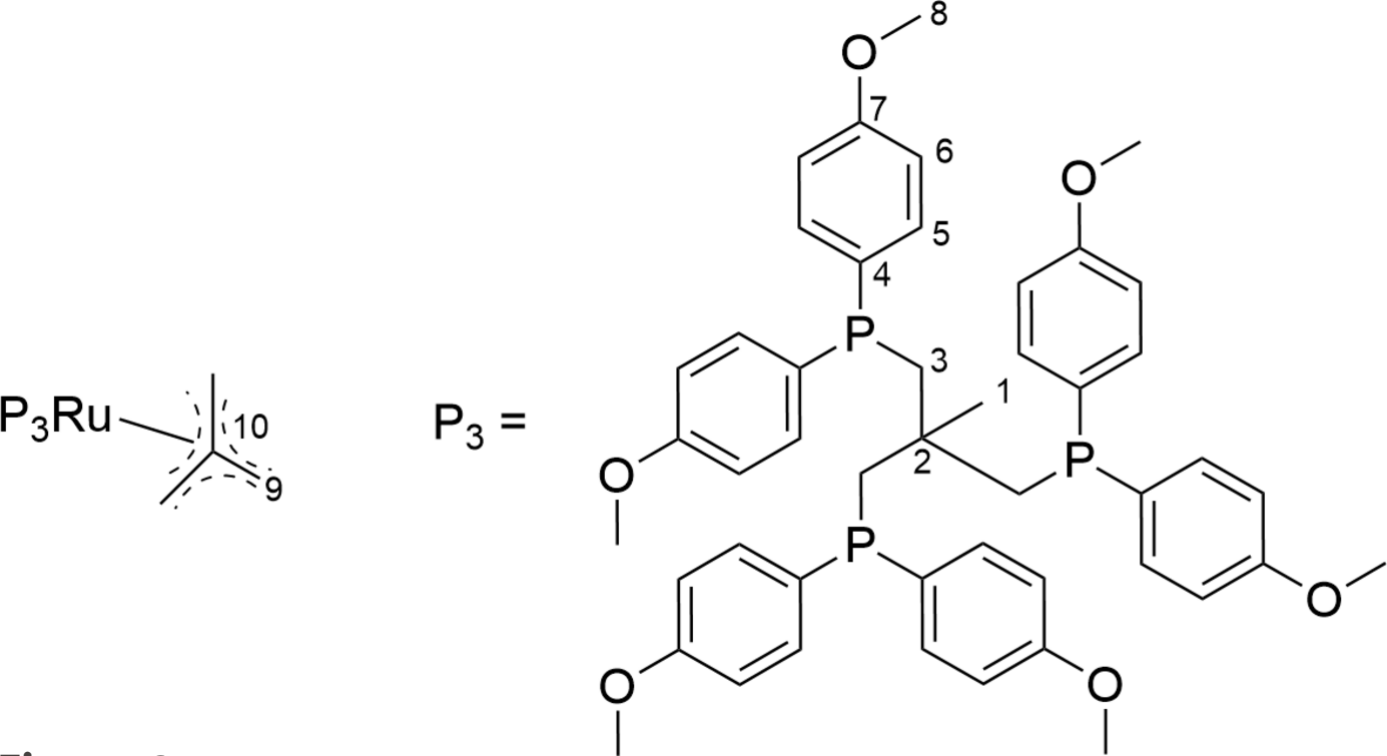
Chemical formula of the title compound with numbering for assignment for the NMR spectra.

^1^H{^31^P}-NMR (300 MHz, [CD_2_Cl_2_]): δ (ppm) = 6.99 (*d*, *J*_HH_ = 9 Hz, H5, 12H), 6.49 (*d*, *J*_HH_ = 9 Hz, H6, 12H), 3.70 (*s*, H8, 18H), 2.14 (*s*, H3, 6H), 1.55 (*s*, H9, 6H), 1.34 (*s*, H1, 3H) (Fig. 2 ▸). ^31^P {^1^H}-NMR (121 MHz, [CD_2_Cl_2_]): δ (ppm) = 31.4 (*s*). ^13^C {^1^H}-NMR (121 MHz, [CD_2_Cl_2_]): δ (ppm) = 159.5 (C7), 134.2 (C4), 133.9 (C5), 113.0 (C6), 106.8 (C10), 55.4 (C8), 42.5 (C9), 39.2 (C1), 38.5 (C2), 36.1 (C3) (see Fig. 2 ▸ for atom numbering).

## Refinement

Crystal data, data collection and structure refinement details are summarized in Table 1 ▸. Atoms H2*A*, H2*B*, H3*A*, H3*B*, H4*A* and H4*B* could be found from the difference-Fourier map and were refined freely. SADI and *DFIX* commands in *SHELXL* were used to optimize the shape of the half occupied diethyl ether mol­ecule. Additionally, SIMU and EADP instructions were included to equalize the displacement parameters of the carbon atoms of the solvent (C53, C54 and C52, C55, respectively). For the final refinement, the contributions of further disordered solvent mol­ecules were removed from the diffraction data with the SQUEEZE procedure in *PLATON* (Spek, 2015 ▸). SQUEEZE estimated the electron counts in the void volume of 328 Å^3^ to be 83.

## Supplementary Material

Crystal structure: contains datablock(s) I. DOI: 10.1107/S2414314625005243/hb4517sup1.cif

Structure factors: contains datablock(s) I. DOI: 10.1107/S2414314625005243/hb4517Isup2.hkl

CCDC reference: 2463507

Additional supporting information:  crystallographic information; 3D view; checkCIF report

## Figures and Tables

**Table 1:** Experimental details

Crystal data	
Chemical formula	[Ru(C_47_H_51_O_6_P_3_)(C_4_H_6_)]·0.5C_4_H_10_O
*M*_r_	997.00
Crystal system, space group	Triclinic, *P* 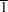
Temperature (K)	130
*a*, *b*, *c* (Å)	9.1504 (13), 11.6521 (16), 24.868 (4)
α, β, γ (°)	95.453 (3), 98.296 (3), 90.372 (3)
*V* (Å^3^)	2611.3 (6)
*Z*	2
Radiation type	Mo *K*α
μ (mm^−1^)	0.44
Crystal size (mm)	0.36 × 0.21 × 0.08

Data collection	
Diffractometer	Bruker APEXII CCD
Absorption correction	Multi-scan (*SADABS*; Krause *et al.*, 2015 ▸)
*T*_min_, *T*_max_	0.86, 0.97
No. of measured, independent and observed [*I* > 2σ(*I*)] reflections	111738, 13249, 12183
*R*_int_	0.033
(sin θ/λ)_max_ (Å^−1^)	0.671

Refinement	
*R*[*F*^2^ > 2σ(*F*^2^)], *wR*(*F*^2^), *S*	0.034, 0.084, 1.10
No. of reflections	13249
No. of parameters	620
No. of restraints	3
H-atom treatment	H atoms treated by a mixture of independent and constrained refinement
Δρ_max_, Δρ_min_ (e Å^−3^)	1.33, −0.71

Computer programs: *APEX2* (Bruker, 2014 ▸), *SAINT* (Bruker, 2013 ▸), *SHELXT* (Sheldrick, 2015*a* ▸), *SHELXL* (Sheldrick, 2015*b* ▸), *XP* in *SHELXTL* (Sheldrick, 2008 ▸) and *ChemDraw* (Mills, 2006 ▸).

## full crystallographic data

### (η4-2-Methylidenepropane-1,3-diyl)(1,1,1-tris{[bis(4-methoxyphenyl)phosphanyl]methyl}ethane)ruthenium(II) diethyl ether hemisolvate Crystal data

**Table:**

[Ru(C_47_H_51_O_6_P_3_)(C_4_H_6_)]·0.5C_4_H_10_O	*Z* = 2
*M**_r_* = 997.00	*F*(000) = 1042
Triclinic, *P*1	*D*_x_ = 1.268 Mg m^−^^3^
*a* = 9.1504 (13) Å	Mo *K*α radiation, λ = 0.71073 Å
*b* = 11.6521 (16) Å	Cell parameters from 9069 reflections
*c* = 24.868 (4) Å	θ = 2.3–29.1°
α = 95.453 (3)°	µ = 0.44 mm^−^^1^
β = 98.296 (3)°	*T* = 130 K
γ = 90.372 (3)°	Needle, pale yellow
*V* = 2611.3 (6) Å^3^	0.36 × 0.21 × 0.08 mm

### (η4-2-Methylidenepropane-1,3-diyl)(1,1,1-tris{[bis(4-methoxyphenyl)phosphanyl]methyl}ethane)ruthenium(II) diethyl ether hemisolvate Data collection

**Table:**

Bruker APEXII CCD diffractometer	13249 independent reflections
Radiation source: fine-focus sealed tube	12183 reflections with *I* > 2σ(*I*)
Detector resolution: 8.3333 pixels mm^-1^	*R*_int_ = 0.033
φ and ω scans	θ_max_ = 28.5°, θ_min_ = 1.7°
Absorption correction: multi-scan (SADABS; Krause *et al.*, 2015)	*h* = −12→12
*T*_min_ = 0.86, *T*_max_ = 0.97	*k* = −15→15
111738 measured reflections	*l* = −33→33

### (η4-2-Methylidenepropane-1,3-diyl)(1,1,1-tris{[bis(4-methoxyphenyl)phosphanyl]methyl}ethane)ruthenium(II) diethyl ether hemisolvate Refinement

**Table:**

Refinement on *F*^2^	Primary atom site location: dual
Least-squares matrix: full	Hydrogen site location: mixed
*R*[*F*^2^ > 2σ(*F*^2^)] = 0.034	H atoms treated by a mixture of independent and constrained refinement
*wR*(*F*^2^) = 0.084	*w* = 1/[σ^2^(*F*_o_^2^) + (0.031*P*)^2^ + 2.8623*P*] where *P* = (*F*_o_^2^ + 2*F*_c_^2^)/3
*S* = 1.10	(Δ/σ)_max_ = 0.002
13249 reflections	Δρ_max_ = 1.33 e Å^−^^3^
620 parameters	Δρ_min_ = −0.71 e Å^−^^3^
3 restraints	

### (η4-2-Methylidenepropane-1,3-diyl)(1,1,1-tris{[bis(4-methoxyphenyl)phosphanyl]methyl}ethane)ruthenium(II) diethyl ether hemisolvate Special details

**Table:**

Geometry. All esds (except the esd in the dihedral angle between two l.s. planes) are estimated using the full covariance matrix. The cell esds are taken into account individually in the estimation of esds in distances, angles and torsion angles; correlations between esds in cell parameters are only used when they are defined by crystal symmetry. An approximate (isotropic) treatment of cell esds is used for estimating esds involving l.s. planes.

### (η4-2-Methylidenepropane-1,3-diyl)(1,1,1-tris{[bis(4-methoxyphenyl)phosphanyl]methyl}ethane)ruthenium(II) diethyl ether hemisolvate Fractional atomic coordinates and isotropic or equivalent isotropic displacement parameters (Å^2^)

**Table:**

	*x*	*y*	*z*	*U*_iso_*/*U*_eq_	Occ. (<1)
C52	0.2204 (13)	0.0943 (7)	0.0211 (6)	0.100 (3)	0.5
H52A	0.321347	0.080457	0.038154	0.150*	0.5
H52B	0.221119	0.108782	−0.017045	0.150*	0.5
H52C	0.181610	0.161598	0.040767	0.150*	0.5
C53	0.1226 (9)	−0.0114 (6)	0.0233 (3)	0.0644 (19)	0.5
H53A	0.161494	−0.079895	0.003776	0.077*	0.5
H53B	0.121860	−0.026913	0.061771	0.077*	0.5
O7	−0.0243 (8)	0.0106 (11)	−0.0018 (11)	0.062 (3)	0.5
C54	−0.1170 (8)	−0.0891 (6)	−0.0001 (3)	0.067 (2)	0.5
H54A	−0.117247	−0.107862	0.037923	0.080*	0.5
H54B	−0.084139	−0.157061	−0.021871	0.080*	0.5
C55	−0.2705 (12)	−0.0508 (8)	−0.0253 (6)	0.100 (3)	0.5
H55A	−0.342776	−0.113620	−0.026021	0.150*	0.5
H55B	−0.299640	0.017133	−0.003268	0.150*	0.5
H55C	−0.266792	−0.031680	−0.062593	0.150*	0.5
C1	0.5470 (2)	0.61076 (18)	0.28588 (8)	0.0242 (4)	
C2	0.4926 (2)	0.65582 (19)	0.33462 (9)	0.0252 (4)	
C3	0.5827 (2)	0.69415 (18)	0.25073 (9)	0.0246 (4)	
C4	0.4727 (2)	0.50839 (18)	0.25813 (9)	0.0263 (4)	
C5	0.3395 (2)	0.66565 (16)	0.11064 (8)	0.0195 (3)	
C6	0.4690 (2)	0.60314 (17)	0.11157 (8)	0.0224 (4)	
H6	0.498324	0.558938	0.141349	0.027*	
C7	0.5568 (2)	0.60328 (17)	0.07037 (8)	0.0238 (4)	
H7	0.644723	0.559989	0.072050	0.029*	
C8	0.5142 (2)	0.66761 (17)	0.02672 (8)	0.0237 (4)	
C9	0.3839 (2)	0.72973 (18)	0.02440 (8)	0.0254 (4)	
H9	0.353865	0.772831	−0.005717	0.030*	
C10	0.2984 (2)	0.72854 (17)	0.06592 (8)	0.0231 (4)	
H10	0.210109	0.771308	0.063997	0.028*	
C11	0.7246 (2)	0.6149 (2)	−0.01589 (9)	0.0327 (5)	
H11A	0.794129	0.643373	0.016441	0.049*	
H11B	0.767124	0.627435	−0.048888	0.049*	
H11C	0.705291	0.532286	−0.015106	0.049*	
C12	0.12986 (19)	0.52183 (16)	0.14134 (7)	0.0190 (3)	
C13	0.1551 (2)	0.42421 (16)	0.16889 (8)	0.0216 (4)	
H13	0.228118	0.427726	0.200326	0.026*	
C14	0.0769 (2)	0.32137 (17)	0.15196 (8)	0.0236 (4)	
H14	0.097120	0.255517	0.171361	0.028*	
C15	−0.0308 (2)	0.31589 (16)	0.10658 (8)	0.0231 (4)	
C16	−0.0551 (2)	0.41133 (17)	0.07690 (8)	0.0246 (4)	
H16	−0.126566	0.406881	0.044977	0.029*	
C17	0.0248 (2)	0.51225 (17)	0.09400 (8)	0.0228 (4)	
H17	0.008361	0.576605	0.073294	0.027*	
C18	−0.1004 (3)	0.12417 (19)	0.11811 (11)	0.0367 (5)	
H18A	−0.000369	0.094808	0.117849	0.055*	
H18B	−0.172719	0.063790	0.101735	0.055*	
H18C	−0.115746	0.146852	0.155818	0.055*	
C19	0.0495 (2)	0.48835 (16)	0.28390 (8)	0.0199 (3)	
C20	0.1490 (2)	0.41319 (18)	0.30955 (10)	0.0291 (4)	
H20	0.247118	0.439675	0.322893	0.035*	
C21	0.1088 (2)	0.30162 (19)	0.31603 (10)	0.0340 (5)	
H21	0.178997	0.252011	0.333241	0.041*	
C22	−0.0350 (2)	0.26201 (17)	0.29728 (9)	0.0274 (4)	
C23	−0.1365 (2)	0.33463 (18)	0.27237 (9)	0.0276 (4)	
H23	−0.234924	0.308154	0.259661	0.033*	
C24	−0.0937 (2)	0.44721 (17)	0.26592 (8)	0.0243 (4)	
H24	−0.164210	0.496784	0.248836	0.029*	
C25	−0.2109 (3)	0.1078 (2)	0.29005 (12)	0.0400 (6)	
H25A	−0.277746	0.155715	0.309584	0.060*	
H25B	−0.217844	0.028018	0.299041	0.060*	
H25C	−0.238896	0.110321	0.250629	0.060*	
C26	0.07060 (19)	0.70110 (16)	0.34557 (8)	0.0197 (3)	
C27	−0.0748 (2)	0.7017 (2)	0.35736 (9)	0.0302 (5)	
H27	−0.152348	0.669662	0.330462	0.036*	
C28	−0.1071 (2)	0.7478 (2)	0.40708 (10)	0.0387 (6)	
H28	−0.206612	0.748902	0.413908	0.046*	
C29	0.0051 (2)	0.7930 (2)	0.44753 (9)	0.0319 (5)	
C30	0.1493 (2)	0.79189 (18)	0.43740 (8)	0.0247 (4)	
H30	0.226816	0.821753	0.464874	0.030*	
C31	0.1803 (2)	0.74652 (16)	0.38648 (8)	0.0213 (4)	
H31	0.279759	0.746755	0.379609	0.026*	
C32	0.0767 (3)	0.8793 (3)	0.53848 (11)	0.0580 (9)	
H32A	0.145836	0.817560	0.547211	0.087*	
H32B	0.033439	0.906693	0.571062	0.087*	
H32C	0.129588	0.943218	0.526316	0.087*	
C33	0.3431 (2)	0.95068 (15)	0.32834 (7)	0.0189 (3)	
C34	0.2506 (2)	1.00783 (17)	0.36094 (8)	0.0218 (4)	
H34	0.147043	1.004400	0.349247	0.026*	
C35	0.3056 (2)	1.07035 (17)	0.41053 (8)	0.0237 (4)	
H35	0.239932	1.108747	0.432187	0.028*	
C36	0.4563 (2)	1.07605 (17)	0.42795 (8)	0.0234 (4)	
C37	0.5518 (2)	1.02067 (19)	0.39530 (9)	0.0279 (4)	
H37	0.655551	1.025500	0.406651	0.034*	
C38	0.4954 (2)	0.95914 (18)	0.34668 (8)	0.0256 (4)	
H38	0.561361	0.921372	0.324953	0.031*	
C39	0.4289 (3)	1.1922 (2)	0.50943 (9)	0.0389 (5)	
H39A	0.375867	1.251689	0.489557	0.058*	
H39B	0.488466	1.228405	0.542708	0.058*	
H39C	0.357675	1.137463	0.519187	0.058*	
C40	0.37004 (19)	0.96025 (16)	0.22014 (8)	0.0199 (3)	
C41	0.3493 (2)	1.07899 (17)	0.22652 (8)	0.0239 (4)	
H41	0.290308	1.109434	0.252756	0.029*	
C42	0.4119 (2)	1.15405 (17)	0.19579 (8)	0.0250 (4)	
H42	0.396987	1.234686	0.201235	0.030*	
C43	0.4971 (2)	1.10942 (17)	0.15678 (8)	0.0233 (4)	
C44	0.5182 (2)	0.99107 (17)	0.14955 (8)	0.0240 (4)	
H44	0.575041	0.960299	0.122691	0.029*	
C45	0.4567 (2)	0.91808 (16)	0.18132 (8)	0.0204 (4)	
H45	0.474000	0.837695	0.176565	0.025*	
C46	0.5586 (3)	1.29621 (18)	0.13423 (9)	0.0310 (4)	
H46A	0.455742	1.320628	0.128510	0.047*	
H46B	0.614246	1.332507	0.109294	0.047*	
H46C	0.602619	1.319536	0.172036	0.047*	
C47	0.0911 (2)	0.76620 (16)	0.15491 (8)	0.0194 (3)	
H47A	0.139073	0.836920	0.145940	0.023*	
H47B	0.019320	0.737444	0.122684	0.023*	
C48	−0.03391 (19)	0.69010 (16)	0.23091 (8)	0.0189 (3)	
H48A	−0.065966	0.627272	0.201836	0.023*	
H48B	−0.119203	0.708473	0.250353	0.023*	
C49	0.08936 (19)	0.89055 (16)	0.24598 (8)	0.0197 (3)	
H49A	0.043689	0.893617	0.279826	0.024*	
H49B	0.077100	0.966839	0.231636	0.024*	
C50	0.00576 (19)	0.79829 (15)	0.20347 (7)	0.0181 (3)	
C51	−0.1412 (2)	0.84997 (17)	0.18005 (8)	0.0230 (4)	
H51A	−0.194292	0.878298	0.210030	0.034*	
H51B	−0.201531	0.790550	0.156104	0.034*	
H51C	−0.121562	0.914016	0.159085	0.034*	
O1	0.58996 (17)	0.67523 (14)	−0.01606 (6)	0.0308 (3)	
O2	−0.11837 (17)	0.22147 (13)	0.08752 (7)	0.0313 (3)	
O3	−0.06470 (18)	0.14986 (13)	0.30570 (8)	0.0381 (4)	
O4	−0.03867 (18)	0.8359 (2)	0.49571 (7)	0.0530 (6)	
O5	0.52291 (16)	1.13276 (14)	0.47578 (6)	0.0309 (3)	
O6	0.56350 (18)	1.17425 (13)	0.12387 (6)	0.0309 (3)	
P1	0.23330 (5)	0.65646 (4)	0.16726 (2)	0.01707 (9)	
P2	0.11526 (5)	0.63599 (4)	0.27960 (2)	0.01704 (9)	
P3	0.28904 (5)	0.86405 (4)	0.26319 (2)	0.01700 (9)	
Ru1	0.34344 (2)	0.67321 (2)	0.25595 (2)	0.01663 (4)	
H2A	0.462 (3)	0.604 (2)	0.3568 (9)	0.022 (6)*	
H2B	0.537 (3)	0.728 (2)	0.3517 (10)	0.025 (6)*	
H3A	0.626 (3)	0.763 (2)	0.2679 (10)	0.030 (6)*	
H3B	0.615 (3)	0.669 (2)	0.2170 (11)	0.033 (7)*	
H4A	0.499 (3)	0.480 (2)	0.2226 (10)	0.025 (6)*	
H4B	0.444 (3)	0.455 (2)	0.2785 (10)	0.025 (6)*	

### (η4-2-Methylidenepropane-1,3-diyl)(1,1,1-tris{[bis(4-methoxyphenyl)phosphanyl]methyl}ethane)ruthenium(II) diethyl ether hemisolvate Atomic displacement parameters (Å^2^)

**Table:**

	*U*^11^	*U*^22^	*U*^33^	*U*^12^	*U*^13^	*U*^23^
C52	0.125 (8)	0.045 (5)	0.112 (5)	−0.021 (4)	−0.050 (5)	0.019 (5)
C53	0.081 (5)	0.057 (4)	0.053 (4)	0.026 (4)	−0.002 (4)	0.005 (3)
O7	0.073 (7)	0.040 (5)	0.072 (4)	−0.004 (5)	0.011 (8)	−0.001 (5)
C54	0.069 (4)	0.056 (4)	0.073 (5)	−0.024 (3)	0.026 (4)	−0.033 (3)
C55	0.125 (8)	0.045 (5)	0.112 (5)	−0.021 (4)	−0.050 (5)	0.019 (5)
C1	0.0125 (8)	0.0307 (10)	0.0281 (10)	0.0060 (7)	−0.0010 (7)	0.0009 (8)
C2	0.0166 (8)	0.0313 (10)	0.0261 (10)	−0.0004 (7)	−0.0026 (7)	0.0035 (8)
C3	0.0104 (8)	0.0298 (10)	0.0317 (10)	0.0018 (7)	0.0025 (7)	−0.0053 (8)
C4	0.0235 (9)	0.0264 (10)	0.0286 (10)	0.0086 (8)	0.0021 (8)	0.0029 (8)
C5	0.0175 (8)	0.0197 (8)	0.0206 (8)	−0.0004 (7)	0.0023 (7)	−0.0014 (7)
C6	0.0207 (9)	0.0234 (9)	0.0229 (9)	0.0016 (7)	0.0025 (7)	0.0018 (7)
C7	0.0200 (9)	0.0256 (9)	0.0261 (9)	0.0044 (7)	0.0042 (7)	0.0020 (7)
C8	0.0231 (9)	0.0263 (9)	0.0215 (9)	−0.0011 (7)	0.0046 (7)	−0.0003 (7)
C9	0.0270 (10)	0.0266 (10)	0.0225 (9)	0.0035 (8)	0.0027 (7)	0.0039 (8)
C10	0.0200 (9)	0.0240 (9)	0.0247 (9)	0.0033 (7)	0.0017 (7)	0.0013 (7)
C11	0.0255 (10)	0.0430 (13)	0.0318 (11)	0.0051 (9)	0.0106 (9)	0.0041 (9)
C12	0.0152 (8)	0.0205 (8)	0.0206 (8)	0.0006 (6)	0.0025 (6)	−0.0009 (7)
C13	0.0184 (8)	0.0231 (9)	0.0225 (9)	0.0038 (7)	0.0008 (7)	0.0007 (7)
C14	0.0235 (9)	0.0198 (9)	0.0274 (10)	0.0024 (7)	0.0019 (7)	0.0045 (7)
C15	0.0208 (9)	0.0198 (9)	0.0278 (10)	−0.0019 (7)	0.0033 (7)	−0.0014 (7)
C16	0.0229 (9)	0.0244 (9)	0.0242 (9)	−0.0017 (7)	−0.0031 (7)	0.0009 (7)
C17	0.0221 (9)	0.0206 (9)	0.0248 (9)	−0.0001 (7)	−0.0007 (7)	0.0037 (7)
C18	0.0373 (12)	0.0237 (10)	0.0479 (14)	−0.0070 (9)	0.0001 (10)	0.0077 (10)
C19	0.0169 (8)	0.0197 (8)	0.0227 (9)	0.0010 (7)	0.0027 (7)	0.0005 (7)
C20	0.0180 (9)	0.0237 (10)	0.0436 (12)	0.0001 (7)	−0.0035 (8)	0.0056 (9)
C21	0.0247 (10)	0.0254 (10)	0.0505 (14)	0.0037 (8)	−0.0027 (9)	0.0092 (9)
C22	0.0267 (10)	0.0195 (9)	0.0361 (11)	−0.0017 (7)	0.0046 (8)	0.0033 (8)
C23	0.0199 (9)	0.0284 (10)	0.0329 (11)	−0.0063 (8)	−0.0013 (8)	0.0033 (8)
C24	0.0179 (9)	0.0256 (9)	0.0287 (10)	0.0002 (7)	−0.0003 (7)	0.0049 (8)
C25	0.0351 (12)	0.0267 (11)	0.0588 (16)	−0.0100 (9)	0.0092 (11)	0.0044 (10)
C26	0.0150 (8)	0.0214 (8)	0.0224 (9)	0.0013 (6)	0.0031 (7)	−0.0001 (7)
C27	0.0140 (8)	0.0447 (12)	0.0291 (10)	−0.0012 (8)	0.0018 (7)	−0.0097 (9)
C28	0.0146 (9)	0.0615 (16)	0.0360 (12)	−0.0008 (9)	0.0049 (8)	−0.0173 (11)
C29	0.0217 (10)	0.0448 (13)	0.0266 (10)	0.0021 (9)	0.0033 (8)	−0.0097 (9)
C30	0.0181 (8)	0.0306 (10)	0.0234 (9)	−0.0009 (7)	−0.0012 (7)	−0.0011 (8)
C31	0.0150 (8)	0.0249 (9)	0.0243 (9)	0.0009 (7)	0.0027 (7)	0.0045 (7)
C32	0.0285 (12)	0.104 (3)	0.0331 (13)	−0.0007 (14)	0.0012 (10)	−0.0324 (15)
C33	0.0169 (8)	0.0187 (8)	0.0209 (8)	−0.0008 (6)	0.0033 (7)	0.0002 (7)
C34	0.0160 (8)	0.0253 (9)	0.0233 (9)	0.0013 (7)	0.0025 (7)	−0.0006 (7)
C35	0.0215 (9)	0.0264 (9)	0.0229 (9)	0.0022 (7)	0.0055 (7)	−0.0023 (7)
C36	0.0230 (9)	0.0252 (9)	0.0210 (9)	−0.0023 (7)	0.0018 (7)	−0.0008 (7)
C37	0.0158 (8)	0.0354 (11)	0.0304 (10)	−0.0026 (8)	0.0020 (7)	−0.0062 (8)
C38	0.0171 (8)	0.0315 (10)	0.0275 (10)	−0.0008 (7)	0.0061 (7)	−0.0051 (8)
C39	0.0349 (12)	0.0517 (15)	0.0265 (11)	0.0042 (10)	0.0027 (9)	−0.0128 (10)
C40	0.0155 (8)	0.0223 (9)	0.0216 (9)	0.0005 (6)	0.0023 (7)	0.0017 (7)
C41	0.0231 (9)	0.0225 (9)	0.0268 (10)	0.0053 (7)	0.0071 (7)	0.0005 (7)
C42	0.0264 (9)	0.0204 (9)	0.0289 (10)	0.0050 (7)	0.0058 (8)	0.0029 (7)
C43	0.0209 (9)	0.0242 (9)	0.0253 (9)	−0.0007 (7)	0.0043 (7)	0.0037 (7)
C44	0.0219 (9)	0.0251 (9)	0.0257 (9)	0.0020 (7)	0.0075 (7)	−0.0002 (7)
C45	0.0165 (8)	0.0197 (8)	0.0246 (9)	0.0001 (6)	0.0033 (7)	−0.0009 (7)
C46	0.0396 (12)	0.0238 (10)	0.0308 (11)	0.0004 (9)	0.0071 (9)	0.0056 (8)
C47	0.0171 (8)	0.0190 (8)	0.0212 (9)	0.0025 (6)	−0.0005 (7)	0.0021 (7)
C48	0.0118 (7)	0.0200 (8)	0.0243 (9)	0.0008 (6)	0.0006 (6)	0.0026 (7)
C49	0.0137 (8)	0.0191 (8)	0.0256 (9)	0.0024 (6)	0.0024 (7)	−0.0009 (7)
C50	0.0138 (7)	0.0191 (8)	0.0214 (8)	0.0025 (6)	0.0013 (6)	0.0028 (7)
C51	0.0162 (8)	0.0221 (9)	0.0305 (10)	0.0031 (7)	0.0011 (7)	0.0048 (7)
O1	0.0283 (7)	0.0407 (9)	0.0265 (7)	0.0080 (6)	0.0109 (6)	0.0083 (6)
O2	0.0307 (8)	0.0221 (7)	0.0381 (9)	−0.0074 (6)	−0.0048 (6)	0.0027 (6)
O3	0.0315 (8)	0.0214 (7)	0.0608 (11)	−0.0036 (6)	0.0020 (8)	0.0082 (7)
O4	0.0213 (8)	0.0959 (16)	0.0343 (9)	−0.0015 (9)	0.0035 (7)	−0.0315 (10)
O5	0.0249 (7)	0.0406 (9)	0.0240 (7)	−0.0013 (6)	0.0011 (6)	−0.0096 (6)
O6	0.0388 (8)	0.0234 (7)	0.0341 (8)	0.0016 (6)	0.0155 (7)	0.0059 (6)
P1	0.01360 (19)	0.0177 (2)	0.0192 (2)	0.00138 (16)	0.00080 (16)	0.00047 (17)
P2	0.01133 (19)	0.0185 (2)	0.0206 (2)	0.00093 (16)	0.00078 (16)	0.00072 (17)
P3	0.01225 (19)	0.0183 (2)	0.0201 (2)	0.00043 (16)	0.00272 (16)	−0.00046 (17)
Ru1	0.01074 (7)	0.01885 (7)	0.01943 (7)	0.00181 (5)	0.00065 (5)	−0.00034 (5)

### (η4-2-Methylidenepropane-1,3-diyl)(1,1,1-tris{[bis(4-methoxyphenyl)phosphanyl]methyl}ethane)ruthenium(II) diethyl ether hemisolvate Geometric parameters (Å, º)

**Table:**

C52—C53	1.529 (8)	C25—O3	1.410 (3)
C52—H52A	0.9800	C25—H25A	0.9800
C52—H52B	0.9800	C25—H25B	0.9800
C52—H52C	0.9800	C25—H25C	0.9800
C53—O7	1.434 (11)	C26—C31	1.385 (3)
C53—H53A	0.9900	C26—C27	1.403 (3)
C53—H53B	0.9900	C26—P2	1.8425 (19)
O7—C54	1.441 (12)	C27—C28	1.373 (3)
C54—C55	1.540 (8)	C27—H27	0.9500
C54—H54A	0.9900	C28—C29	1.392 (3)
C54—H54B	0.9900	C28—H28	0.9500
C55—H55A	0.9800	C29—O4	1.369 (3)
C55—H55B	0.9800	C29—C30	1.378 (3)
C55—H55C	0.9800	C30—C31	1.393 (3)
C1—C2	1.431 (3)	C30—H30	0.9500
C1—C3	1.433 (3)	C31—H31	0.9500
C1—C4	1.435 (3)	C32—O4	1.438 (3)
C1—Ru1	2.0674 (18)	C32—H32A	0.9800
C2—Ru1	2.245 (2)	C32—H32B	0.9800
C2—H2A	0.92 (2)	C32—H32C	0.9800
C2—H2B	0.96 (2)	C33—C34	1.385 (2)
C3—Ru1	2.2249 (18)	C33—C38	1.402 (3)
C3—H3A	0.93 (3)	C33—P3	1.8275 (19)
C3—H3B	0.95 (3)	C34—C35	1.397 (3)
C4—Ru1	2.263 (2)	C34—H34	0.9500
C4—H4A	0.97 (2)	C35—C36	1.383 (3)
C4—H4B	0.90 (2)	C35—H35	0.9500
C5—C6	1.393 (3)	C36—O5	1.364 (2)
C5—C10	1.398 (3)	C36—C37	1.397 (3)
C5—P1	1.8335 (19)	C37—C38	1.376 (3)
C6—C7	1.390 (3)	C37—H37	0.9500
C6—H6	0.9500	C38—H38	0.9500
C7—C8	1.389 (3)	C39—O5	1.423 (3)
C7—H7	0.9500	C39—H39A	0.9800
C8—O1	1.360 (2)	C39—H39B	0.9800
C8—C9	1.396 (3)	C39—H39C	0.9800
C9—C10	1.383 (3)	C40—C45	1.392 (3)
C9—H9	0.9500	C40—C41	1.395 (3)
C10—H10	0.9500	C40—P3	1.8435 (19)
C11—O1	1.422 (3)	C41—C42	1.387 (3)
C11—H11A	0.9800	C41—H41	0.9500
C11—H11B	0.9800	C42—C43	1.395 (3)
C11—H11C	0.9800	C42—H42	0.9500
C12—C13	1.387 (3)	C43—O6	1.366 (2)
C12—C17	1.404 (3)	C43—C44	1.392 (3)
C12—P1	1.8398 (19)	C44—C45	1.383 (3)
C13—C14	1.390 (3)	C44—H44	0.9500
C13—H13	0.9500	C45—H45	0.9500
C14—C15	1.383 (3)	C46—O6	1.422 (3)
C14—H14	0.9500	C46—H46A	0.9800
C15—O2	1.363 (2)	C46—H46B	0.9800
C15—C16	1.394 (3)	C46—H46C	0.9800
C16—C17	1.379 (3)	C47—C50	1.548 (3)
C16—H16	0.9500	C47—P1	1.8448 (18)
C17—H17	0.9500	C47—H47A	0.9900
C18—O2	1.422 (3)	C47—H47B	0.9900
C18—H18A	0.9800	C48—C50	1.554 (3)
C18—H18B	0.9800	C48—P2	1.8456 (18)
C18—H18C	0.9800	C48—H48A	0.9900
C19—C24	1.389 (3)	C48—H48B	0.9900
C19—C20	1.397 (3)	C49—C50	1.549 (3)
C19—P2	1.8365 (19)	C49—P3	1.8504 (18)
C20—C21	1.379 (3)	C49—H49A	0.9900
C20—H20	0.9500	C49—H49B	0.9900
C21—C22	1.391 (3)	C50—C51	1.537 (2)
C21—H21	0.9500	C51—H51A	0.9800
C22—O3	1.374 (2)	C51—H51B	0.9800
C22—C23	1.381 (3)	C51—H51C	0.9800
C23—C24	1.397 (3)	P1—Ru1	2.2787 (6)
C23—H23	0.9500	P2—Ru1	2.2988 (5)
C24—H24	0.9500	P3—Ru1	2.2780 (6)
			
C53—C52—H52A	109.5	C29—C30—H30	120.3
C53—C52—H52B	109.5	C31—C30—H30	120.3
H52A—C52—H52B	109.5	C26—C31—C30	122.06 (17)
C53—C52—H52C	109.5	C26—C31—H31	119.0
H52A—C52—H52C	109.5	C30—C31—H31	119.0
H52B—C52—H52C	109.5	O4—C32—H32A	109.5
O7—C53—C52	108.6 (10)	O4—C32—H32B	109.5
O7—C53—H53A	110.0	H32A—C32—H32B	109.5
C52—C53—H53A	110.0	O4—C32—H32C	109.5
O7—C53—H53B	110.0	H32A—C32—H32C	109.5
C52—C53—H53B	110.0	H32B—C32—H32C	109.5
H53A—C53—H53B	108.3	C34—C33—C38	117.36 (17)
C53—O7—C54	108.9 (12)	C34—C33—P3	127.20 (14)
O7—C54—C55	103.0 (8)	C38—C33—P3	115.44 (14)
O7—C54—H54A	111.2	C33—C34—C35	121.78 (17)
C55—C54—H54A	111.2	C33—C34—H34	119.1
O7—C54—H54B	111.2	C35—C34—H34	119.1
C55—C54—H54B	111.2	C36—C35—C34	119.62 (18)
H54A—C54—H54B	109.1	C36—C35—H35	120.2
C54—C55—H55A	109.5	C34—C35—H35	120.2
C54—C55—H55B	109.5	O5—C36—C35	125.08 (18)
H55A—C55—H55B	109.5	O5—C36—C37	115.32 (17)
C54—C55—H55C	109.5	C35—C36—C37	119.61 (18)
H55A—C55—H55C	109.5	C38—C37—C36	119.85 (18)
H55B—C55—H55C	109.5	C38—C37—H37	120.1
C2—C1—C3	115.95 (18)	C36—C37—H37	120.1
C2—C1—C4	115.50 (18)	C37—C38—C33	121.77 (18)
C3—C1—C4	114.71 (18)	C37—C38—H38	119.1
C2—C1—Ru1	77.48 (11)	C33—C38—H38	119.1
C3—C1—Ru1	76.55 (11)	O5—C39—H39A	109.5
C4—C1—Ru1	78.20 (11)	O5—C39—H39B	109.5
C1—C2—Ru1	64.03 (10)	H39A—C39—H39B	109.5
C1—C2—H2A	117.9 (15)	O5—C39—H39C	109.5
Ru1—C2—H2A	116.4 (15)	H39A—C39—H39C	109.5
C1—C2—H2B	115.6 (14)	H39B—C39—H39C	109.5
Ru1—C2—H2B	113.6 (14)	C45—C40—C41	117.67 (17)
H2A—C2—H2B	118 (2)	C45—C40—P3	121.63 (14)
C1—C3—Ru1	64.65 (10)	C41—C40—P3	120.68 (14)
C1—C3—H3A	116.2 (16)	C42—C41—C40	122.12 (18)
Ru1—C3—H3A	115.1 (16)	C42—C41—H41	118.9
C1—C3—H3B	119.9 (16)	C40—C41—H41	118.9
Ru1—C3—H3B	117.6 (16)	C41—C42—C43	119.05 (18)
H3A—C3—H3B	114 (2)	C41—C42—H42	120.5
C1—C4—Ru1	63.42 (10)	C43—C42—H42	120.5
C1—C4—H4A	117.9 (14)	O6—C43—C44	115.84 (17)
Ru1—C4—H4A	112.8 (14)	O6—C43—C42	124.51 (18)
C1—C4—H4B	117.9 (16)	C44—C43—C42	119.65 (18)
Ru1—C4—H4B	117.1 (15)	C45—C44—C43	120.27 (18)
H4A—C4—H4B	117 (2)	C45—C44—H44	119.9
C6—C5—C10	117.52 (17)	C43—C44—H44	119.9
C6—C5—P1	117.64 (14)	C44—C45—C40	121.22 (18)
C10—C5—P1	124.82 (14)	C44—C45—H45	119.4
C7—C6—C5	122.20 (18)	C40—C45—H45	119.4
C7—C6—H6	118.9	O6—C46—H46A	109.5
C5—C6—H6	118.9	O6—C46—H46B	109.5
C8—C7—C6	119.06 (18)	H46A—C46—H46B	109.5
C8—C7—H7	120.5	O6—C46—H46C	109.5
C6—C7—H7	120.5	H46A—C46—H46C	109.5
O1—C8—C7	124.95 (18)	H46B—C46—H46C	109.5
O1—C8—C9	115.14 (18)	C50—C47—P1	114.60 (12)
C7—C8—C9	119.91 (18)	C50—C47—H47A	108.6
C10—C9—C8	120.04 (18)	P1—C47—H47A	108.6
C10—C9—H9	120.0	C50—C47—H47B	108.6
C8—C9—H9	120.0	P1—C47—H47B	108.6
C9—C10—C5	121.27 (18)	H47A—C47—H47B	107.6
C9—C10—H10	119.4	C50—C48—P2	115.84 (12)
C5—C10—H10	119.4	C50—C48—H48A	108.3
O1—C11—H11A	109.5	P2—C48—H48A	108.3
O1—C11—H11B	109.5	C50—C48—H48B	108.3
H11A—C11—H11B	109.5	P2—C48—H48B	108.3
O1—C11—H11C	109.5	H48A—C48—H48B	107.4
H11A—C11—H11C	109.5	C50—C49—P3	114.06 (12)
H11B—C11—H11C	109.5	C50—C49—H49A	108.7
C13—C12—C17	117.35 (17)	P3—C49—H49A	108.7
C13—C12—P1	119.79 (14)	C50—C49—H49B	108.7
C17—C12—P1	122.87 (14)	P3—C49—H49B	108.7
C12—C13—C14	122.08 (18)	H49A—C49—H49B	107.6
C12—C13—H13	119.0	C51—C50—C47	107.08 (15)
C14—C13—H13	119.0	C51—C50—C49	107.66 (15)
C15—C14—C13	119.33 (18)	C47—C50—C49	112.30 (15)
C15—C14—H14	120.3	C51—C50—C48	106.66 (14)
C13—C14—H14	120.3	C47—C50—C48	111.73 (15)
O2—C15—C14	124.54 (18)	C49—C50—C48	111.08 (15)
O2—C15—C16	115.53 (18)	C50—C51—H51A	109.5
C14—C15—C16	119.93 (18)	C50—C51—H51B	109.5
C17—C16—C15	119.84 (18)	H51A—C51—H51B	109.5
C17—C16—H16	120.1	C50—C51—H51C	109.5
C15—C16—H16	120.1	H51A—C51—H51C	109.5
C16—C17—C12	121.37 (18)	H51B—C51—H51C	109.5
C16—C17—H17	119.3	C8—O1—C11	117.95 (17)
C12—C17—H17	119.3	C15—O2—C18	116.94 (17)
O2—C18—H18A	109.5	C22—O3—C25	117.59 (18)
O2—C18—H18B	109.5	C29—O4—C32	116.31 (18)
H18A—C18—H18B	109.5	C36—O5—C39	116.73 (17)
O2—C18—H18C	109.5	C43—O6—C46	117.42 (16)
H18A—C18—H18C	109.5	C5—P1—C12	98.09 (8)
H18B—C18—H18C	109.5	C5—P1—C47	102.81 (8)
C24—C19—C20	117.37 (18)	C12—P1—C47	102.11 (8)
C24—C19—P2	124.96 (14)	C5—P1—Ru1	121.90 (6)
C20—C19—P2	117.60 (14)	C12—P1—Ru1	117.49 (6)
C21—C20—C19	121.86 (19)	C47—P1—Ru1	111.63 (6)
C21—C20—H20	119.1	C19—P2—C26	98.09 (9)
C19—C20—H20	119.1	C19—P2—C48	101.82 (8)
C20—C21—C22	119.74 (19)	C26—P2—C48	101.90 (8)
C20—C21—H21	120.1	C19—P2—Ru1	121.79 (6)
C22—C21—H21	120.1	C26—P2—Ru1	118.98 (6)
O3—C22—C23	124.68 (19)	C48—P2—Ru1	111.21 (6)
O3—C22—C21	115.52 (19)	C33—P3—C40	97.02 (9)
C23—C22—C21	119.80 (19)	C33—P3—C49	104.08 (8)
C22—C23—C24	119.71 (18)	C40—P3—C49	101.80 (9)
C22—C23—H23	120.1	C33—P3—Ru1	119.19 (6)
C24—C23—H23	120.1	C40—P3—Ru1	118.98 (6)
C19—C24—C23	121.51 (18)	C49—P3—Ru1	113.02 (6)
C19—C24—H24	119.2	C1—Ru1—C3	38.80 (8)
C23—C24—H24	119.2	C1—Ru1—C2	38.49 (8)
O3—C25—H25A	109.5	C3—Ru1—C2	65.82 (8)
O3—C25—H25B	109.5	C1—Ru1—C4	38.37 (8)
H25A—C25—H25B	109.5	C3—Ru1—C4	65.11 (8)
O3—C25—H25C	109.5	C2—Ru1—C4	65.06 (8)
H25A—C25—H25C	109.5	C1—Ru1—P3	123.22 (6)
H25B—C25—H25C	109.5	C3—Ru1—P3	97.30 (6)
C31—C26—C27	117.30 (17)	C2—Ru1—P3	102.46 (6)
C31—C26—P2	121.24 (14)	C4—Ru1—P3	161.15 (6)
C27—C26—P2	121.40 (14)	C1—Ru1—P1	126.73 (6)
C28—C27—C26	121.20 (19)	C3—Ru1—P1	104.12 (6)
C28—C27—H27	119.4	C2—Ru1—P1	164.85 (6)
C26—C27—H27	119.4	C4—Ru1—P1	100.79 (6)
C27—C28—C29	120.39 (19)	P3—Ru1—P1	89.759 (17)
C27—C28—H28	119.8	C1—Ru1—P2	129.19 (6)
C29—C28—H28	119.8	C3—Ru1—P2	167.20 (6)
O4—C29—C30	124.57 (19)	C2—Ru1—P2	101.69 (6)
O4—C29—C28	115.76 (19)	C4—Ru1—P2	107.93 (6)
C30—C29—C28	119.67 (19)	P3—Ru1—P2	87.931 (17)
C29—C30—C31	119.35 (18)	P1—Ru1—P2	87.511 (19)
